# Women with suspected diagnosis of ovarian cancer in Ghana: how much do we know about them?

**DOI:** 10.3389/fonc.2026.1771387

**Published:** 2026-03-12

**Authors:** Kwabena Amo-Antwi, Yvonne Nartey, Ramatu Agambire, Lauren Davis-Rivera, Roxanna Haghighat, Bernard Worlasi Ocloo, Adu Appiah-Kubi, Philip Agyemang-Prempeh, George Osei Prempeh, Kofi Dekyi, Ama Yeboah Boakye, Edward Tieru Dassah, Elliot Koranteng Tannor, Mavis Bobie Ansah, Patrick Kafui Akakpo, Ernest Adjei, Nana Addo Boateng, Kofi Nti Maxwell, Kwabena Oppong Adutwum, Kwasi Ampem-Darkwa, Bismark Dwobeng Amo, Akwasi Antwi-Kusi, Eloise Chapman-Davis

**Affiliations:** 1 Department of Obstetrics and Gynaecology, School of Medical Sciences, Kwame Nkrumah University of Science and Technology, Kumasi, Ghana; 2 Walter Aiden Specialist Clinic, Asokwa-Kumasi, Ghana; 3 Department of Obstetrics and Gynaecology, Komfo Anokye Teaching Hospital, Kumasi, Ghana; 4 Department of Adult Health, School of Nursing and Midwifery, University of Ghana, Accra, Ghana; 5 Department of Nursing and Midwifery, Garden City University College, Kumasi, Ghana; 6 Department of Obstetrics and Gynaecology, Weill Cornell Medical Centre, New York-Presbyterian, New York, NY, United States; 7 Biostatics Unit, Komfo Anokye Teaching Hospital, Kumasi, Ghana; 8 Department of Obstetrics and Gynaecology, School of Medicine, University of Health and Allied Sciences, Ho, Ghana; 9 Department of Population, Family and Reproductive Health, School of Public Health, Kwame Nkrumah University of Science and Technology, Kumasi, Ghana; 10 Department of Medicine, School of Medical Sciences, Kwame Nkrumah University of Science and Technology, Kumasi, Ghana; 11 Department of Pathology, School of Medical Sciences, University of Cape Coast, Cape Coast Teaching Hospital, Cape Coast, Ghana; 12 Department of Pathology, Komfo Anokye Teaching Hospital, Kumasi, Ghana; 13 Department of Oncology, Komfo Anokye Teaching Hospital, Kumasi, Ghana; 14 Department of Anaesthesiology and Intensive Care, Komfo Anokye Teaching Hospital, Kumasi, Ghana

**Keywords:** clinical characteristics, demographic characteristics, epithelial ovarian cancers, germ cell tumours, Ghana, ovarian cancer, sex cord-stromal tumours, sub-Saharan Africa

## Abstract

**Background:**

Addressing disparities in ovarian cancer care between sub-Saharan Africa and other regions begins with the fundamental question, “Who gets ovarian cancer?” This study aimed to identify the demographic and clinical predictors of cancer among women with ovarian tumours.

**Methods:**

We conducted a cross-sectional analysis of women with histology-confirmed ovarian tumour discussed at a multidisciplinary tumour board in a tertiary hospital in Ghana between 2013 and 2024. Descriptive statistics were used to summarise the characteristics of the women, and logistic regression was performed to identify predictors of ovarian cancer. P≤0.05 was considered statistically significant.

**Results:**

Of the 496 women whose data were analysed, 74.4% (n=369) had ovarian cancer. Women diagnosed with ovarian cancer were older than those without cancer (median age 50 years (36-60) vs 39 years (26-55), p<0.001). Most women (274, 55.3%) were married; 297 (59.9%) were multiparous; 291 (58.7%) were urbanites; and more women with cancer were unemployed (93 vs 8, p=0.023). Most women (281, 56.7%) reported abdominal distension. Women with cancer had anaemia (p<0.001), hypertension, diabetes, or other comorbidities (p=0.012). Of the 127 benign tumours, mature cystic teratomas (50, 39.4%), mucinous tumours (28, 22.0%), serous tumours (20, 15.7%), and ovarian fibromas (12, 12.9%) were common. Few (30, 7.5%) and 3 (0.8%) had immunohistochemical and genetic tests, respectively. Travel distance of 100 km or more, (aOR 2.82; 95% CI: 1.21–6.56) postmenopausal status (aOR 6.43; 95% CI: 1.98–20.92), symptom duration of 3–6 months (aOR 2.85; 95% CI: 1.36–5.95), anaemia (aOR 3.74; 95% CI: 1.93–7.25) and hypertension, diabetes, or other comorbidities (aOR 3.74; 95% CI: 1.93–7.25) were predictors of ovarian cancer. Other predictors were tumours with a solid component (aOR 12.97; 95% CI: 3.16–53.18), vascular flow on imaging (aOR 9.53; 95% CI: 4.63–19.65), ascites (aOR 3.62; 95% CI: 1.84–7.14) and elevated serum CA-125 levels (aOR 4.48; 95% CI: 2.32–8.62).

**Conclusion:**

Most women with ovarian tumours were young, and a significant proportion had benign tumours, highlighting the need for a more thorough diagnostic assessment. Improved gynaecological ultrasound scanning, access to intraoperative pathology consultation and molecular testing will be essential for guiding ovarian cancer care in Ghana.

## 1 Introduction

Ovarian cancer is a major cause of morbidity and mortality among women globally, accounting for over 300,000 new cases and more than 200,000 deaths worldwide each year (1). In Ghana, ovarian cancer ranks as the third most prevalent cancer in women (2). As in many other settings, survival outcomes are significantly associated with the stage at diagnosis. Yet women with ovarian cancer often present late, frequently with advanced disease (3). This is largely due to its insidious onset, nonspecific early symptoms and the absence of effective population-based screening strategies. The differential diagnosis, therefore, spans a wide spectrum of benign, borderline, and malignant ovarian tumours to other abdominopelvic conditions masquerading as ovarian tumours (4). The objective of preoperative assessment in women with an ovarian tumour is to accurately identify and manage cases of malignant disease, while avoiding unnecessary surgical interventions or overtreatment of benign tumours.

The epidemiology of ovarian cancer in African populations differs from that observed in Western countries, including variations in incidence, histological distribution and age at presenation (5–8). Women with a suspected diagnosis of the disease are often in their forties and fifties, and the diagnostic challenge extends beyond differentiating malignant from benign ovarian tumours, but also distinguishing them from other conditions, such as abdominal tuberculosis, degenerated leiomyoma, and endometriosis, all of which may mimic ovarian cancer. A family history of cancer, especially breast or ovarian cancer, in first- or second-degree relatives, represents the most significant risk (9). Advanced age is recognised as a risk factor, as most ovarian cancers are diagnosed in postmenopausal women (10). Increasing parity appears to protect against ovarian cancer, but it is uncertain how much this association is exhibited among younger West African women with ovarian tumours (11).

Access to specialist care from diagnostic workup, through surgical staging, histopathological confirmation of cancer, to availability of systemic chemotherapy and targeted agents, varies widely across countries and even within regions of the same country (12). A symptomatic woman residing in rural areas often face long travel distances to obtain specialist care, and this increased travel burden to a treatment centre or rural residence correlates with delayed diagnosis. Marital status may influence the decision to seek care, often mediated by spousal assistance in the form of supportive care during treatment at the specialist centre (13). Higher educational attainment improves health literacy, while women who are employed are more likely to seek care promptly (14).

In the absence of an effective population-based screening method, clinical assessment remains central to the evaluation of women with a suspected diagnosis of ovarian cancer in settings where advanced diagnostic pathways are limited. A new-onset abdominal distension or mass, associated with pain, easy satiety or nausea, dominates ovarian cancer symptoms (15, 16). The duration of symptoms varies between women with and without the disease. An ultrasound of the abdomen or pelvis, used alone or in conjunction with associated algorithms, can differentiate between benign and malignant tumours, but critical expertise is required (17, 18).

A significant increase in the risk of ovarian cancer has been reported in women with conditions such as hypertension, diabetes, obesity, hyperlipidaemia and non-alcoholic fatty liver disease (19). Anaemia is present in more than 30% of patients with epithelial ovarian cancer at initial presentation (20).

Primary curative or maximal-effort ovarian debulking surgery, followed by platinum-based chemotherapy and appropriate maintenance treatment, yields favourable outcomes with manageable morbidity for ovarian malignancies (21). Advances in precision medicine driven by biomarker-guided treatment, have enhanced outcomes and transformed ovarian cancer management globally (22). Mutations in the breast cancer genes 1 & 2 (BRCA1 and BRCA2), along with alterations in other homologous recombination repair genes, serve as key prognostic biomarkers in ovarian cancer care. The growing array of actionable biomarkers has introduced new therapeutic opportunities, including the development of antibody-drug conjugates that offer meaningful survival benefits, even for patients who are ineligible for platinum-based therapy. Addressing the disparities in ovarian cancer care between sub-Saharan Africa and other regions begins with the fundamental question, “Who gets ovarian cancer?”. Generating context-specific data is essential for informing clinical practice, enhancing early recognition, and supporting the development of context-appropriate diagnostic algorithms. Additionally, characterising the profile of women who are ultimately diagnosed with ovarian cancer can contribute to improved clinician awareness and strengthen patient education. This study aimed to identify the demographic and clinical predictors of cancer among women with ovarian tumours using routinely collected data at the Komfo Anokye Teaching Hospital (KATH), Kumasi, Ghana.

## 2 Materials and methods

### 2.1 Study design

We conducted a retrospective analysis of data of women with histology-confirmed ovarian tumours presented at the multidisciplinary tumour board (Gynae Onc Tumour Board) at a major referral hospital in Ghana, from January 2013 to December 2024.

### 2.2 Study site

The research was conducted at the KATH, specifically within the Departments of Obstetrics, Gynaecology and Oncology. KATH, situated in the Ashanti region of Ghana, is the country’s second-largest hospital and functions as a referral centre for 13 of the 16 administrative regions in Ghana. In 2023, there were 291 confirmed cases of gynaecological cancers, comprising 165 cervical, 72 ovarian, 32 uterine, 7 vulvar cancers, and 26 cases of choriocarcinoma. The treatment protocol is determined individually by the Gynae Onc Tumour Board and frequently aligns with the National Comprehensive Cancer Network (NCCN) protocols.

At initial presentation, all women with ovarian tumour underwent a standard clinical assessment, which included an ultrasound scan of the pelvis and abdomen, as well as a chest X-ray. A request for tumour markers is often done serially, starting with CA-125 and proceeding to additional markers as indicated. Computed tomography (CT) and/or magnetic resonance imaging (MRI) are requested entirely based on the patient’s ability to afford the scan. The standard surgical treatment for suspected ovarian cancer comprised total abdominal hysterectomy (TAH), bilateral salpingo-oophorectomy (BSO) with appropriate surgical staging, which often included peritoneal washing, omentectomy, and peritoneal biopsies with or without lymph node dissection. The scope of surgical intervention is determined by the pre-operative findings and the surgeon’s evaluation of the tumour’s macroscopic characteristics. For tumours grossly confined to an ovary, the presence of excrescence, nodules, and papillae on the tumour wall upon bisection may also dictate the extent of the surgery. Fertility-sparing ovarian surgery was defined as salpingo-ophorectomy, performed with or without surgical staging.

### 2.3 Data source

**Figure 1:**
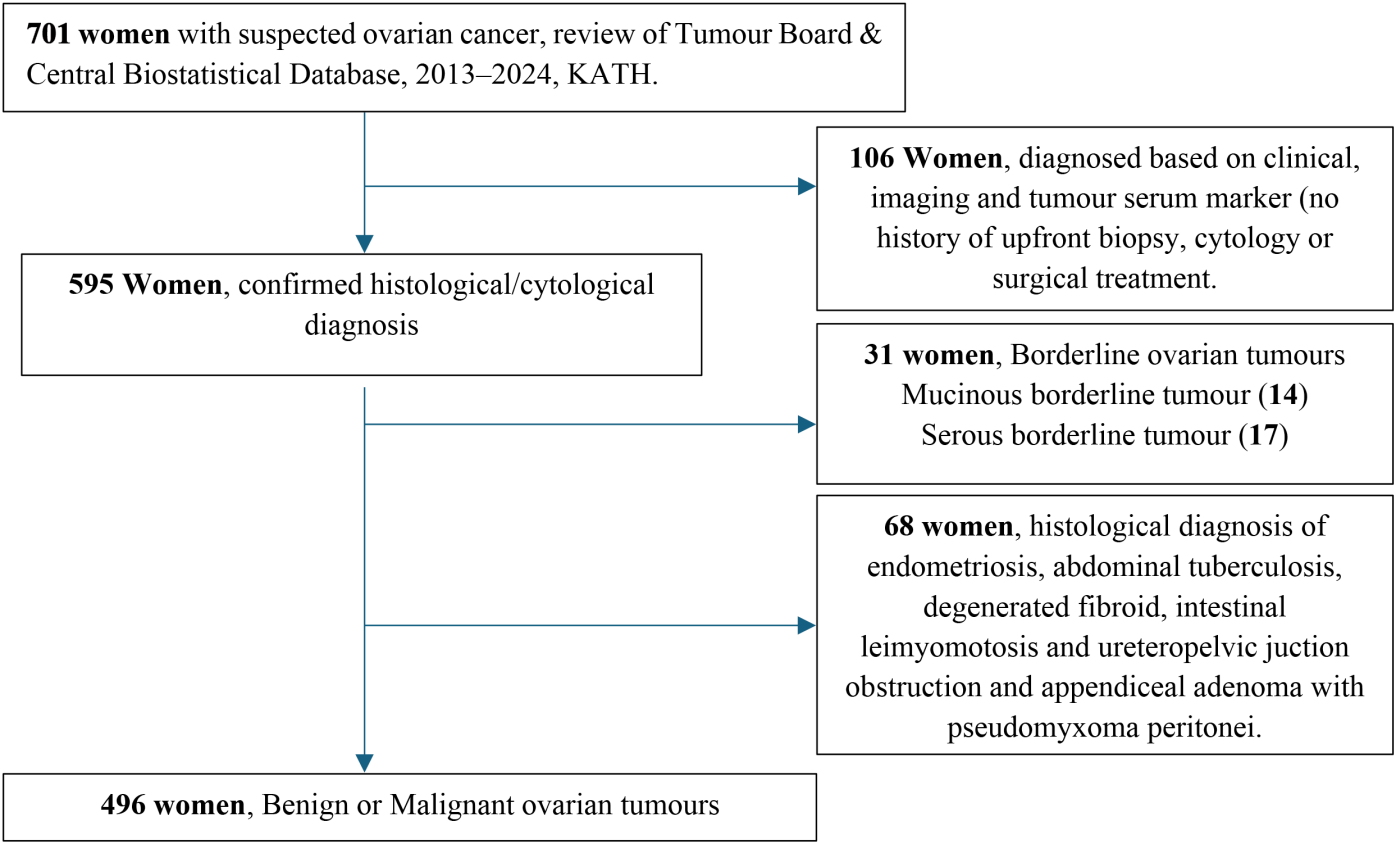
Flow chart of the source of data of women with suspected diagnosis of ovarian cancer.

The monthly Gynae Onco Tumour Board maintains demographic, clinical, pathological, and treatment records for all women with suspected diagnoses of ovarian cancer. The demographic, clinical, and treatment data were independently extracted and de-identified by two trainee fellows in gynaecologic oncology into a standardised format. Data accuracy and prevention of duplication were achieved through the interdepartmental linkage of the following variables: unique identification, age, telephone contact, and histology report number. The principal investigator conducted daily checks for the completeness of the collected data. The majority of missing data were on the survival status (alive or deceased) of cancer patients who were lost to follow-up. In these cases, patients or their relatives were contacted for missing data following verbal consent. Borderline ovarian tumours exhibit diagnostic imaging features and serum levels of tumour markers, such as CA-125, that are similar to those of malignant ovarian tumours (23). The differentiation of malignant ovarian tumours from benign ovarian histotypes based on clinical characteristics may be undermined by the inclusion of borderline tumours in either category (23). For this reason, data of women with borderline ovarian tumours were excluded. A total of 595 women were identified with ovarian tumours, following biopsies (percutaneous or laparotomy) or definitive surgeries (primary curative, primary or interval debulking surgery) (**Figure 1**).

### 2.4 Statistical analysis

We summarised participants’ demographic, clinical and surgical data using descriptive statistics. Frequencies and percentages were used for categorical variables and means with standard deviations or medians with interquartile ranges (IQR) for continuous variables. Chi-square or Fisher’s exact tests were used for categorical variables, while Student’s t-test was utilised for continuous variables, to evaluate associations between variables and ovarian cancer diagnosis. To examine predictors of ovarian cancer, we first conducted univariate logistic regression analyses for individual clinical and diagnostic parameters. Variables with p-values < 0.25 in the univariate analysis were included in a multivariate logistic regression model to identify independent predictors, while adjusting for potential confounders. Statistical significance was set at p < 0.05. All analyses were performed using Stata^®^ version 17.0 (StataCorp, College Station, TX, USA).

### 2.5 Ethical approval

The research protocol, questionnaire, and consent statement received approval from the Institutional Review Board and Research & Development Unit of Komfo Anokye Teaching Hospital (KATH/IRB/CA/167/25), as well as from the Committee on Human Research, Publications and Ethics at Kwame Nkrumah University of Science and Technology (CHRPE/AP/1030/25). It is the standard practice to collect contact information for all women diagnosed with ovarian cancer to facilitate patient navigation and minimise treatment defaults or loss to follow-up. In instances of missing data for the study, patients or their next of kin were contacted by telephone, and informed verbal consent was obtained from the respondents before the interview. Both institutional review boards approved this approach, consistent with methods used in previous studies by Karasik et al. (24) and Amo-Antwi et al. (25).

## 3 Results

### 3.1 Demographic data

A total of 496 women, with a median age of 48 years (IQR 31-59), were analysed (**Table 1**). Women diagnosed with ovarian cancer exhibited a significantly higher median age of 50 years (IQR 36-60) compared to 39 years (IQR 26-55) in the benign group (p<0.001). Most women with ovarian tumour were premenopausal, 273 (55%). However, a significant proportion of women with cancer were postmenopausal (184 vs 39, p<0.001). Seventeen women (3.4%) with cancer had a family history of cancer, compared to zero for those without cancer. Most of the women (274, 55.3%) were married, 297 (59.9%) had two or more children, 58.7% (n=291) resided in urban areas, and about a quarter (n=124) travelled 100 km or more to reach the treatment centre. Among the women studied, significantly more women, 93 (25.4%), diagnosed with the cancer were unemployed (p=0.023).

**Table 1:** Demographic data.

Characteristics, Number^a^ (n=496)	Total, n (%)	Malignant, n (%)	Benign, n (%)	P-value
Age group (years)				< 0.001
Median (IQR)	48 (31-59)	50 (36-60)	39 (26-55)	
< 30	108 (21.8)	67 (18.2)	41 (32.3)	
30-44	101 (20.4)	69 (18.7)	32 (25.2)	
45-59	164 (33.0)	131 (35.5)	33 (26.0)	
>/=60	123 (24.8)	102 (27.6)	21 (16.5)	
BMI (Kg/m^2^)				0.494
Median (IQR)	24.6 (22.0-29.4)	24.9 (22.0-29.1)	24.2 (21.6-29.5)	
< 18.5	49 (9.9)	37 (10.0)	12 (9.5)	
18.5-24.9	215 (43.4)	149 (40.4)	66 (52.0)	
25.0-29.5	115 (23.2)	96 (26.0)	19 (15.0)	
>30	117 (23.6)	87 (23.6)	30 (23.6)	
Menopausal status				< 0.001
Post-menopausal	223 (45.0)	184 (49.9)	39 (30.7)	
Premenopausal	273 (55.0)	185 (50.1)	88 (69.3)	
Parity				0.130
Median (IQR)	2 (1-4)	2 (1-4)	2 (0-4)	
0	116 (23.4)	81 (22.0)	35 (27.6)	
1	83 (16.7)	59 (16.0)	24 (18.9)	
2-4	193 (38.9)	150 (40.7)	43 (33.9)	
5+	104 (21.0)	79 (21.3)	25 (19.7)	
Residence				0.523
Urban	291 (58.7)	215 (58.3)	76 (59.8)	
Rural	205 (41.3)	154 (41.7)	51 (40.2)	
Travel distance (Km)				0.150
Median (IQR)	16 (9.2-97.5)	20(9.5-121)	14 (9.0-62.0)	
<50	304 (61.3)	212 (57.5)	92 (72.4)	
50-99	68 (13.7)	52 (14.1)	16 (12.6)	
100+	124 (25.0)	105 (28.5)	19 (15.0)	
Level of education				0.138
No formal education	143 (28.8)	115 (31.2)	28 (22.1)	
Primary	162 (32.7)	120 (32.5)	42 (33.1)	
Junior/Senior High	134 (27.0)	93 (25.2)	41 (32.3)	
Tertiary	57 (11.5)	41 (11.1)	16 (12.6)	
Occupation				0.023
Formal	80 (16.1)	42 (11.4)	38 (29.9)	
Informal	315 (63.5)	234 (63.4)	81 (63.8)	
Unemployed	101 (20.4)	93 (25.2)	8 (6.3)	
Marital status				
Married	274 (55.3)	204 (55.3)	70 (55.1)	0.412
Single	135 (27.2)	94 (25.5)	41 (32.3)	
Divorced/Widow	87 (17.5)	71 (19.2)	16 (12.6)	

a :values are given as number unless otherwise stated. IQR, interquartile range; BMI, Body mass index; Kg/m2, kilogram per metre square; Km, kilometre.

### 3.2 Diagnostic data

Abdominal distension or mass was observed in 430 (86.7%) of the symptomatic women (**Table 2**). Notably, women with cancer reported a higher number of secondary complaint (62 versus 9, p=0.01). Of the women studied, 349 (70.4%) were anaemic, and a significant proportion were women with cancer (277 vs. 72, p<0.001). Close to a third, 145 individuals had a comorbid condition, mainly hypertension or diabetes, a significantly higher number in those with cancer (119 vs. 26, p<0.001). A greater number of women with cancer presented tumours with a solid component (268 vs. 108, p<0.001), tumours demonstrating flow under Doppler interrogation (172 vs. 21, p<0.001), or ascites (174 vs. 31, p<0.001). In the studied population, 342 (72.9%) had a CA-125 level of 35 U/ml or higher. Women with cancer exhibited a median serum CA-125 level of 226.5 U/ml (IQR 57-789.0), which was significantly higher than the median level of 31.6 (IQR 17.1-66.5) observed in the benign cohort.

**Table 2:** Diagnostic data.

Characteristics, Number^a^ (n=496)	Total, n (%)	Malignant, n (%)	Benign, n (%)	P-value
Main symptom				0.575
Abdominal Distension	281 (56.7)	220 (59.6)	61 (48.0)	
Abdomino-pelvic mass	149 (30.0)	97 (26.3)	52 (40.9)	
Abdominal pain	46 (9.3)	35 (9.5)	11 (8.7)	
Vaginal Bleeding/urinary retention	20 (4.0)	17 (4.6)	3 (3.8)	
Symptom duration (Months)				< 0.001
Median (IQR)	5 (3-8)	5 (3-7)	6 (3-9)	
< 3	83 (16.7)	62 (16.8)	21 (16.5)	
3–6	267 (53.8)	214 (58.0)	53 (41.7)	
6+	146 (29.4)	93 (25.2)	53 (41.7)	
Secondary compliant				
No	425 (85.7)	307 (83.2)	118 (92.9)	0.007
Yes	71 (14.3)	62 (16.8)	9 (7.1)	
Family history of cancer				0.009*
Yes	17 (3.4)	17 (4.6)	0 (0.0)	
No	479 (96.6)	352 (95.4)	127 (100.0)	
Anaemia, g/dL				< 0.001
Median (IQR)	11.2 (9.8-12.2)	10.9 (9.7-12.0)	11.8 (11.2-12.5)	
Non-anaemic	147 (29.6)	92 (24.9)	55 (43.3)	
Anaemic	349 (70.4)	277 (75.1)	72 (56.7)	
Comorbidities				0.012
No	351 (70.7)	250 (67.8)	101 (79.5)	
Yes	145 (29.3)	119 (32.3)	26 (20.5)	
No of comorbidities, N = 154				
1	121 (83.5)	100 (84.0)	21 (80.8)	0.779
1+	24 (16.5)	19 (16.0)	5 (19.2)	
Solid component				< 0.001
No	23 (5.8)	4 (1.5)	19 (15.0)	
Yes	376 (94.2)	268 (98.5)	108 (85.0)	
Doppler flow				< 0.001
No	206 (51.6)	100 (36.8)	106 (83.5)	
Yes	193 (48.4)	172 (63.2)	21 (16.5)	
Ascites				< 0.001
No	194 (48.6)	98 (36.0)	96 (75.6)	
Yes	205 (51.4)	174 (64.0)	31 (24.4)	
Cross sectional imaging				0.077
Yes	97 (19.6%)	79 (21.4%)	18 (14.2%)	
No	399 (80.4%)	290 (78.6%)	109 (85.8%)	
Serum CA-125, IU/ml				0.001
Median (IQR)	113 (33.1-543)	226.5 (57.0-789.0)	31.6 (17.1-66.5)	
< 35	127 (27.1)	58 (16.8)	69 (55.7)	
35+	342 (72.9)	287 (83.2)	55 (44.3)	

a :values are given as number unless otherwise stated; IQR: interquartile range, *Fisher Exact test.

### 3.3 Surgical data

Three hundred and eighty-four women (77.4%) underwent definitive surgery for ovarian tumours, and 112 (22.6%) received upfront biopsy, all within the malignant cohort (p<0.001) (**Table 3**). A total of 129 women (33.6%) underwent uterus-sparing surgery, with a significantly higher proportion in the benign group (26.3% vs 48.8%, p=0.01). The overall complication rate was 3.2%, with rates of 3.6% for the malignant group and 2.4% for the benign group (p=0.772). In the benign group, 22 women underwent pelvic lymph node dissection (PLND), para-aortic lymph node dissection (PALND), or both, alongside unilateral salpingo-oophorectomy (USO) or TAH, with one case resulting in an injury to the inferior vena cava during lymph node dissection.

**Table 3:** Surgical data.

Characteristics, Number^a^ (n=496)	Total, n (%)	Malignant, n (%)	Benign, n (%)	P-value
Surgical Intervention				
Biopsy alone	112 (22.6)	112 (30.4)	0 (0.0)	< 0.001
Primary surgery	384 (77.4)	257 (69.6)	127 (100.0)	
Definitive surgery n=384				0.009
USO+/-OMT	114 (29.7)	58 (22.6)	56 (44.1)	
TAH_USO/BSO+/-OMT	189 (49.2)	140 (54.5)	49 (38.6)	
USO+/-OMT+LND	15 (3.9)	9 (3.7)	6 (4.7)	
TAH_BSO-/+OMT+LND	66 (17.2)	50 (19.5)	16 (12.6)	
Lymph node dissection n=81				
PLND Alone	37 (45.7)	26 (44.1)	11 (50.0)	0.913*
PLND & PALND	40 (49.4)	30 (50.9)	10 (45.4)	
PALND Alone	4 (4.9)	3 (5.1)	1 (4.6)	
Complications				
Yes	16 (3.2)	13 (3.5)	3 (2.4)	0.772*
No	480 (96.6)	356 (96.5)	124 (97.6)	
Type of complication n=16				
Bladder injury	7 (43.8)	7 (53.9)	0 (0.0)	0.098*
Bowel injury	6 (37.5)	4 (30.8)	2 (66.7)	
Uretric injury	2 (12.5)	2 (15.4)	0 (0.0)	
Vascular injury (IVC)	1 (6.2)	0 (0.0)	1 (33.3)	

a :values are given as number unless otherwise stated. USO, Unilateral salpingo-oophorectomy; OMT, omentectomy or omental biopsy; TAH, total abdominal hysterectomy; BSO, Bilateral salpingo-oophorectomy; PLND, pelvic lymph dissection; PALND, para-aortic lymph dissection; IVC, inferior vena cava. *Fisher Exact test.

### 3.4 Predictors of ovarian cancer among symptomatic women

In the multivariable logistic regression model, women who travelled 100 km or more were more likely to have the cancer (aOR 2.82; 95% CI: 1.21–6.56) (**Table 4**). Likewise, the diagnosis of ovarian cancer was significantly associated with postmenopausal status (aOR 6.43; 95% CI: 1.98–20.92), a symptom duration of 3 to 6 months (aOR 2.85; 95% CI: 1.36–5.95), anaemia (aOR 3.904; 95% CI: 1.93-7.34), and the presence of hypertension, diabetes or other comorbid conditions (aOR 3.74; 95% CI: 1.93–7.25). Tumours with solid components (aOR 12.97; 95% CI: 3.16–53.18), vascular flow on imaging (aOR 9.53; 95% CI: 4.63–19.65), the presence of ascites (aOR 3.62; 95% CI: 1.84–7.14), and elevated serum CA-125 levels (aOR 4.48; 95% CI: 2.32–8.62).

**Table 4:** Univariable and multivariable logistic regression analysis: predictors of ovarian cancer.

Characteristics	Cancers n^a^ (%)	OR*	Univariable	p-value	OR†	Multivariable	P-value
			95% CI			95% CI	
Age, years							
<30	67 (18.2)	1			1		
30-44	69 (18.7)	1.319	0.75-2.34	0.342	0.679	0.26-1.74	0.423
45-59	131 (35.5)	2.429	1.41-4.19	0.001	0.81I9	0.28-2.39	0.715
60+	102 (27.6)	2.972	1.62-5.47	<0.001	0.563	0.12-2.68	0.470
Travel distance (km)							
<50	212 (57.5)	1			1		
50-99	52 (14.1)	1.41	0.76-2.60	0.270	2.046	0.74-5.22	0.172
100+	105 (28.5)	2.398	1.39-4.14	0.002	2.820	1.21-6.56	0.016
Symptom duration, (months)							
6+	62 (16.8)	1			1		
3-6	214 (58.0)	2.301	1.46-3.61	<0.001	2.848	1.36-5.95	0.005
< 3	93 (25.2)	1.683	0.92-3.06	0.089	2.849	0.84-5.34	0.113
Menopausal status							
Pre-menopause	185 (50.1)	1			1		
Post-menopause	184 (49.9)	2.244	1.46-3.45	<0.001	6.432	1.98-20.92	0.002
Haemoglobin level, g/dL							
Non-anaemic	92 (24.9)	1			1		
Anaemia	277 (75.1)	2.3	1.51-3.51	<0.001	4.345	2.17-8.70	< 0.001
Comorbidities							
No	250 (67.8)	1			1		
Yes	119 (32.3)	1.849	1.14-3.00	0.013	2.495	1.10-5.67	0.029
Nature of tumour							
Solid component							
No	4 (1.5)	1			1		
Yes	268 (98.5)	11.787	3.92-35.45	0.0001	11.253	2.67-47.51	0.001
Doppler_flow							
No	100 (36.8)	1			1		
Yes	172 (63.2)	8.682	5.11-14.73	0.0001	14.026	6.54-30.08	< 0.001
Ascites							
No	98 (36.0)	1			1		
Yes	174 (64.0)	5.498	3.24-8.25	0.0001	4.154	2.13-8.12	< 0.001
CA-125 level, U/ml							
< 35	58 (16.8)	1			1		
35+	287 (83.2)	6.208	4.90-12.24	0.0001	4.014	2.05-7.87	< 0.001

a :values are given as number unless otherwise stated. OR*, Crude odd ratio; OR†, Adjusted odds ratio; CI, confidence interval; Stepwise regression, variables with p≥0.25 were not included in the multivariable regression model; km, kilometre; g/dL, gram/decilitre; U/ml, units/ml.

### 3.5 Pathological data

Among the symptomatic women studied, 369 (74.4%) had a malignant ovarian tumour, including 199 (53.9%) with epithelial ovarian cancer, followed by sex cord stromal tumours in 73 (19.8%) and germ cell tumours in 58 (15.7%) (**Table 5**). Among the cohort with malignant ovarian cancer, 212 women, representing 65.2%, had FIGO stage III or IV disease. The five most common primary benign ovarian tumours in women with a suspected diagnosis of ovarian cancer were mature cystic teratoma (50; 39.4%), followed by mucinous cystadenoma (28; 22.0%), serous cystadenoma (20; 15.7%), ovarian fibroma (12; 9.4%), and benign Brenner’s tumour (5; 3.9%). Very few women, 30 (8.1%) and 4 (1.1%), underwent immunohistochemical and genetic testing, respectively.

**Table 5:** Pathological data.

Parameter	Number^a^ (N = 496)	Percentage (%)
Tumour behaviour groups		
Benign ovarian tumours	127	25.6
Malignant ovarian tumours	369	74.4
Tumour groups (WHO)		
Epithelial ovarian tumours	254	51.2
Mesenchymal tumours	1	0.2
Sex cord stromal tumours	90	18.1
Germ cell tumours	108	21.8
Miscellaneous	38	7.7
Tumour like lesions	5	1.0
Benign ovarian tumours, N = 127		
Mature cystic teratoma	50	39.4
Mucinous cystadenoma	28	22.0
Serous cystadenoma	20	15.7
Mucinous adenofibroma	1	0.7
Serous adenofibroma	1	0.7
Fibroma	12	9.4
Benign Brenner’s tumour	5	3.9
Follicular/Luteal cyst	5	3.9
Thecoma	3	2.4
Sclerosing sex cord stromal tumour	2	1.6
Malignant ovarian tumours, N = 369		
Epithelial ovarian cancer	199	53.9
Sex cord stromal tumours	73	19.8
Germ cell tumours	58	15.7
Others/Miscellaneous	39	10.6
Metastatic adenocarcinoma to ovarian	31	8.4
Neuroendocrine tumours of the ovary	1	0.2
Lymphoma	3	0.8
Small cell carcinoma ovary, hypercalcaemic type	2	0.5
Leimyosracoma	1	0.2
EGIST	1	0.2
	Number^a^ (N = 496)	Percentage (%)
Immunohistochemistry test		
Yes	30	8.1
No	339	91.9
Genetic testing		
Yes	3	0.8
No	366	99.2
FIGO stage, N = 325		
Stage IA/B	81	24.9
Stage IC	18	5.5
Stage II	14	4.3
Stage III	70	21.5
Stage IV	142	43.7

a :values are given as number unless otherwise stated.: FIGO, International Federation of Gynaecologist and Obstetricsl; WHO, World health organization; MGCT, mixed germ cell tumours; YST, yolk sac tumour; IT, immature teratoma; DYS, dysgerminoma; CHORIO, choriocarcinoma; EGIST, extra-gestational stromal tumour.

## 4 Discussion

Women diagnosed with ovarian cancer in the cohort were often older, likely post-menopausal, unemployed, and often travelled more than 100 km to access specialist care. Compared with those with benign ovarian tumours, more women presented with abdominal distension and were likely to report a secondary complaint, had a shorter duration of symptoms, a family history of cancer, anaemia, or cardiometabolic conditions. Ultrasound markers of malignancy and elevated serum CA-125 levels were common in women with cancer diagnoses. Notably, a small number of women with benign disease underwent unnecessary radical surgery, due to the persistent pre-operative and intra-operative diagnostic challenges in ovarian cancer diagnosis.

The cancer occurring in the older age group indicated that age is a significant predictor. This is consistent with the established correlation between advancing age and heightened ovarian cancer risk (10). The median age of 50 years for women with cancer aligns with prior studies in the sub-region and supports the predominance of epithelial ovarian cancer (5–8). Epithelial ovarian cancers constituted the largest histological subgroup, agrees with global patterns, although their proportional dominance was less marked than that reported in high-income settings (26).

The relatively higher contribution of sex cord–stromal and germ cell tumours likely reflects the younger age structure of the population and underscores important regional differences in ovarian tumour epidemiology. The overall proportion of malignant cases in our cohort was approximately three times higher than rates reported in a previous Ghanaian study (8). Several reasons may be adduced for this observation. Unlike earlier research, that largely relied mainly on histological specimens, the current study incorporated clinical and treatment data, potentially improving diagnostic capture. The higher cancer incidence observed in our cohort may be attributable to improved referral patterns following the establishment of the first in-country gynaecologic oncology training programme at KATH (27). The improved logistics and human resource capacity for managing gynaecologic oncologic cases likely resulted in the centre receiving a disproportionate number of clinically advanced ovarian cancer cases. In contrast, women with an early-stage disease or who have limited access to care may never present at the specialist centre.

Although most women were premenopausal, which is expected for a young population, those diagnosed with cancer were largely postmenopausal, supporting menopause as a predictor for ovarian cancer (28). The relatively young and predominantly married, is typically associated with higher fecundity. The prevalence of women with higher parity in both cohorts contradicts the protective effect of increased parity on ovarian cancer risk. Increasing parity has historically been associated with a reduced ovarian cancer risk due to the frequent suppression of ovulation (11). This association in African populations remains insufficiently characterised (29). Population-based studies will be essential to elucidate the true magnitude of reproductive, environmental, and tumour-specific factors on ovarian cancer risk in our subregion.

Another notable finding was a correlation between long travel distance and the diagnosis of ovarian cancer. Persistent geographic disparities driven by complex human resource, logistics, and infrastructural constraints continue to shape cancer care inequities throughout sub-Saharan Africa (30). Most women with ovarian tumours were urban dwellers, but a quarter travelled a longer distance to access specialist care. As of 2025, only a few centres in Ghana, the Komfo Anokye Teaching, Korle Bu Teaching, Ho Teaching, Battor Catholic Hospitals, the University of Ghana Medical Centre, Walter Aiden Specialist Clinic and the Ghana Swedish Medical Centre, had established varying capacities for ovarian cancer care. However, these centres are located in three cities: two in the southern part and one in the central part of Ghana, highlighting the pronounced regional imbalance in access to specialised services. While centralisation of cancer care improves expertise and outcomes, it should also be designed to minimise travel burden, which can itself become a barrier to timely diagnosis and treatment.

A substantial proportion of women with a strong clinical suspicion of malignancy were ultimately diagnosed with benign tumours, most commonly mature cystic teratomas and cystadenomas, and these women had a lower median age, supporting age as a useful demographic index in the assessment of women with ovarian tumours. While the prevalence of germ cell tumours is an established regional epidemiological trend, another noteworthy observation was the prevalence and numerical correlation between mucinous cystadenoma and mucinous carcinoma. This observation remains speculative at this point since most mucinous carcinomas originate from the gastrointestinal tract and typically outnumber true primary ovarian mucinous tumours (31–33). Previous studies have documented the challenges posed by limited intraoperative pathology support, unnecessary radical procedure, and their associated prolonged operative time and increased perioperative morbidity (34, 35). Intraoperative frozen section, when available has demonstrated excellent accuracy in distinguishing benign from malignant ovarian masses, significantly reducing unnecessary staging surgeries and associated complications (36). Twenty-two women underwent lymph node dissection, and one patient sustained an inferior vena cava (IVC) injury during surgery, yet all these women were ultimately diagnosed with benign ovarian tumours. This highlights the pressing need for more cautious surgical decision-making and strengthened diagnostic pathways to prevent avoidable morbidity. The incorporation of CDX2, SATB2, and PAX8, along with CK7 and CK20 testing, into the standard immunohistochemistry panel for assessing ovarian histotypes, especially in high-grade tumours and rare ovarian cancer subtypes, will be essential for achieving a conclusive histopathological diagnosis. Accurate classification increasingly informs prognosis assessment, the choice of systemic chemotherapy, and eligibility for targeted therapies. Targeted therapies based on an array of actionable biomarkers, including antibody-drug conjugates, have produced moderate survival benefits, even for patients ineligible for platinum-based therapy.

New-onset abdominal distension or pain associated with easy satiety and nausea remains characteristic of ovarian cancer (15, 16). Consistently, most women with ovarian tumours reported abdominal distension, with an increased frequency of this symptom correlating with a higher likelihood of malignancy. This finding likely reflects the predominance of advanced-stage ovarian cancer in our cohort, where peritoneal spread and ascites are present. Additionally, symptom duration of 3–6 months was strongly associated with increased odds of malignancy, suggesting a period of latency for possible disease spread to the peritoneum. Various scoring systems have been used to characterise ovarian masses in the pre-operative period (18, 37). Although ultrasound, whether used alone or combined with diagnostic algorithms, can differentiate between benign and malignant tumours to an extent, its accuracy is highly dependent on the expertise of the person. While solid components, vascular flow, ascites, and elevated CA-125 demonstrated strong associations with malignancy in this cohort, reliance on these parameters alone may reduce diagnostic precision, particularly in early-stage disease or non-epithelial tumours. The association of ultrasound markers in this cohort underscores the diagnostic value of ultrasound, particularly given its widespread availability in low-resource settings. Enhancing training and proficiency in abdominopelvic ultrasound would significantly improve the triage and early detection of ovarian cancers, ultimately strengthening diagnostic pathways where access to advanced imaging is limited.

The high prevalence of anaemia in women with ovarian tumours is higher than the observed rates among survivors of cervical cancer in a previous study in Ghana (25). Complex interactions between tumour cells and the immune system, as well as underlying nutritional factors, may contribute to anaemia in this cohort (38). Overexpression of inflammatory cytokines reduces red blood cell survival, suppresses erythroid progenitor cells, impairs iron utilisation, and reduces erythropoietin synthesis. Anaemia indicates the presence of biologically active tumour cell clones, which makes it a significant clinical indicator of cancer. Ghana, like many sub-Saharan African countries, are experiencing nutrition transitions, characterised by shifts in food habits and physical activity levels associated with an increasing incidence of cardiometabolic conditions (39, 40). Hypertension and diabetes were common among patients with cancer. The rate is relatively low compared to those who were treated for cervical cancer in a previous study in Ghana (41). The low prevalence may be attributed to the generally low median age of women diagnosed with ovarian tumours.

The primary strength of this study is its emphasis on women with clinically suspected ovarian cancer, which accurately reflects the real-world diagnostic hurdles faced in routine cancer care in Ghana. This study presents the largest, well-characterised cohort of women with histology-confirmed ovarian cancer cases in the West Africa sub-region to date. This data provides context-specific, relevant guidance for clinical decision-making and healthcare policy development in Ghana. This study has several limitations. The possibility of selection bias is introduced by the fact that this study was conducted at a single tertiary referral institution. The demographic and clinical data of women who did not visit the teaching hospital restrict the generalizability of the study to the entire population of women with ovarian tumours. The prospectively collected tumour board data were supplemented by retrospective data extraction from clinical records, which may contain incomplete documentation. Although standardised data extraction procedures and independent review were implemented, and telephone interviews were conducted to improve reliability, some degree of misclassification cannot be excluded. Limitations in the utilisation of immunohistochemistry and genetic testing, potentially leading to histotype misclassification, especially in high-grade tumours and rare ovarian cancer subtypes. Borderline ovarian tumours were excluded from the analysis; while this approach enabled a clearer comparison between malignant and benign cases, it may have reduced the ability to capture the full clinical spectrum of ovarian pathology. These limitations require that the findings of this study be interpreted appropriately.

## 5 Conclusion

This study offers significant insights into the demographic and clinical characteristics of women suspected of having ovarian cancer in Ghana. A significant number of women exhibiting clinical features indicative of ovarian malignancy were relatively young, with many ultimately receiving a diagnosis of benign ovarian tumours. Enhanced access to imaging and intra-operative frozen section, and the integration of molecular stratification into standard practice, will improve risk assessment and lead to better treatment outcomes. A more integrated, data-driven approach is crucial for enhancing ovarian cancer care and guiding evidence-based health policies in Ghana. Future research should focus on precisely characterising the burden and patterns of ovarian cancer in Ghana through the utilisation of population-based cancer registries.

## Data Availability

The raw data supporting the conclusions of this article will be made available by the authors, without undue reservation.
